# Video-assisted thoracoscopic sleeve lobectomy via a single intercostal space three-port approach: Case report

**DOI:** 10.1097/MD.0000000000007449

**Published:** 2017-07-07

**Authors:** Lian Wang, Saibo Pan, Ming Wu

**Affiliations:** Department of Thoracic Surgery, the Second Affiliated Hospital, Zhejiang University School of Medicine, Hangzhou, China.

**Keywords:** lung cancer surgery, minimally invasive surgery, postoperative care, rehabilitation, thoracoscopy/VATS

## Abstract

**Introduction:**

Video-assisted thoracoscopic sleeve lobectomy is safe and feasible for lung cancer. We describe a case of video-assisted thoracoscopic sleeve lobectomy via a novel single intercostal space (SIC) three-port approach.

**Conclusions:**

This case demonstrates that a SIC three-port thoracoscopic approach is effective in sleeve lobectomy, and possesses potential advantages in perioperative pain management and rehabilitation.

## Abstract 2

Supplemental Digital Content is available in the text

## 1 Introduction

Video-assisted thoracoscopic surgery (VATS) sleeve lobectomies in the treatment of central lung cancer have been reported.^[1,2]^ However, the published cases were performed by using conventional multiple-port or single incision approach. Here we describe a left upper lobe sleeve lobectomy via a single intercostal space (SIC) three-port VATS.

## 2 Case

**Figure 1:**
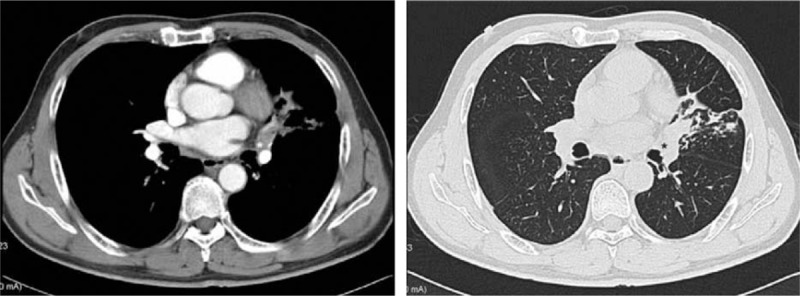
CT scan revealed a central tumor (asterisk) obstructing the left upper lobe bronchus orifice.

A 58 years male was admitted due to cough and blood-tainted sputum since a month. The CT-scan revealed a 1 cm central tumor in the lingular segment with peripheral atelectasis (Fig. 1). Bronchoscopy demonstrated an intraluminal lesion obstructing the left upper lobe bronchus orifice and the biopsy returned a diagnosis of squamous cell carcinoma. Preoperative staging revealed no lymph node involvement or distant metastasis. The pulmonary function test results showed forced expiratory volume in 1 second (FEV_1_) 2.65 L (89%), forced vital capacity (FVC) 3.56 L (95%), and FEV_1_/FVC ratio of 74%. We decided to use a SIC three-port thoracoscopic approach.

**Figure 2:**
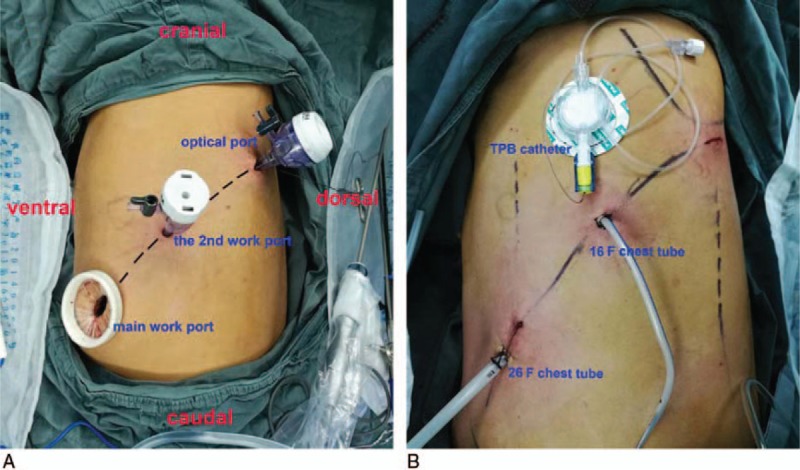
(A) Distribution of incisions. (B) Postoperative chest tubes and TPB catheter of another typical patient.

The patient was placed in the right lateral decubitus position under general anaesthesia supported by epidural anaesthesia, with double lumen endotracheal intubation. All the 3 ports were made in the sixth intercostal space: a 3-cm main work port (the anterior axillary line), a 0.5-cm secondary work port (the median axillary line), and a 1-cm optical port (between the posterior axillary line and subscapular line) (Fig. 2A and Supplemental Video 1). The fissure, pulmonary arteries, and vein were divided using a linear stapler. The left upper bronchus was circumferentially cut off using scissors. The resected lobe was retrieved. During the above process, the lymph node stations 11, 12, 6, 5, 4, 10, and 7 were dissected. Vessel loops were placed to retract the left main pulmonary artery up and backward to ensure adequate surgical exposure of the bronchus. A sleeve resection of the left main bronchus was performed and frozen sections showed negative margins. An end-to-end bronchial anastomosis was performed by simple continuous suturing. We used 3-0 polypropylene running sutures beginning at the posterior wall and maneuvered the stump of the bronchus by an endoscopic forceps. Next, we adjusted the distance between adjacent sutures to ensure an improved matching of proximal to distal bronchus before completing the anterior wall suturing. The anastomosis was then strengthened by interrupted 3-0 single-strand absorbable sutures. Two chest tubes (one 26 F through the main work port and another 16 F through the secondary work port) were placed. The total surgery time was 220 minutes, including 55 minutes for performing the anastomosis. Estimated intraoperative blood loss was 130 mL.

**Figure 3:**
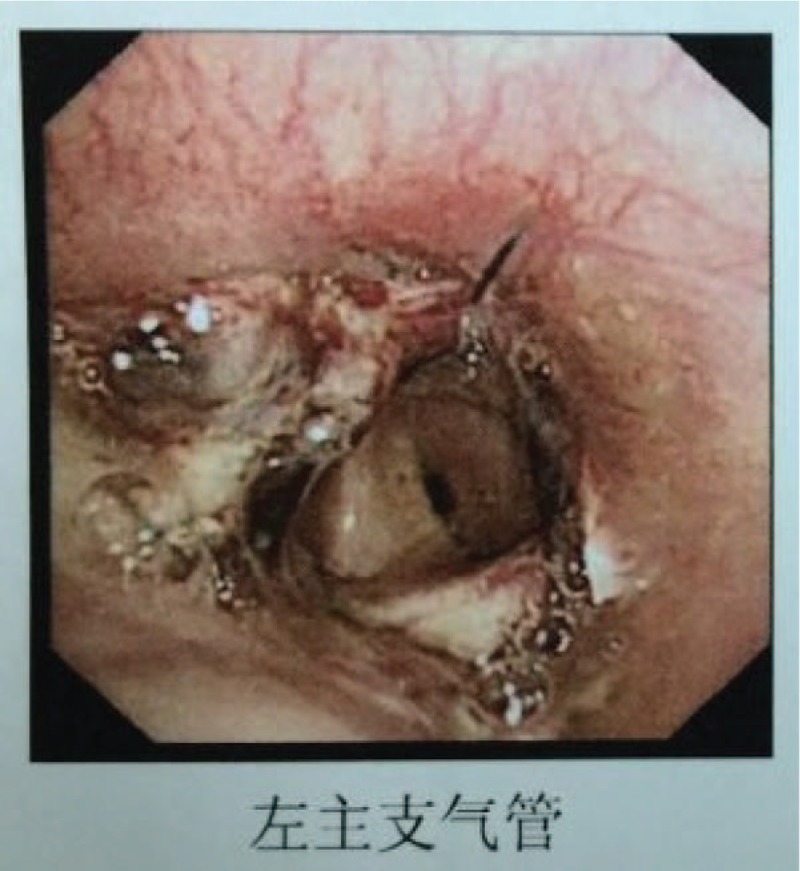
Postoperative bronchoscopy (POD 35).

The larger chest tube and the smaller one were removed on postoperative day (POD) 2 and 5, respectively. He was discharged home on POD 8 with full recovery. The pathological examination revealed no lymph node involvement, and the tumor was classified as pT2aN0M0, stage Ib. The postoperative bronchoscopy confirmed no stenosis (Fig. 3). He was seen at the clinic 2 weeks later with no complaints and only minor discomfort from the incisions (visual analogue scale was rated as 1).

## 3 Comment

A SIC three-port approach may produce less postoperative pain and paresthesia. The arrangement of all incisions in the same intercostal space could theoretically result in better patient tolerability because only one intercostal nerve is irritated and the torque forces on the rib periosteum and intercostal nerve are reduced. Moreover, we usually perform thoracic paravertebral block (TPB) via the optical port to achieve surgical anesthesia (not applied in this case). Because the optical port incision locates relatively close to the vertebral column, it is more reliable to access a 19-G × 100 mm catheter (PlexoLong Nanoline, Pajunk, Inc., Geisingen, Germany) for continuous TPB under direct vision through this incision (Fig. 2B and Supplemental Video 2), without any ultrasound guidance.^[3]^ Compared to the other available regional techniques TPB offers better quality of analgesia and less side effects.^[4]^

A SIC three-port approach could also result in shorter rehabilitation stays. Besides the less nerve damage and excellent pain control, we typically insert 2 chest tubes through the main and the secondary work port for better postoperative drainage. In our surgical practice, the large chest tube connecting to water seal drainage system could be removed in up to two-thirds patients with no air-leak on POD 1. The remaining small, 16 F chest tube with plastic bag is removed in the next few days. This protocol will obviously lead to early mobilization and lung expansion.

The surgeon and first assistant stand on the ventral and dorsal side of the patient, respectively. A 10-mm, 30° thoracoscope provides accurate visualization of the thoracic structures. Furthermore, all the procedures were performed using widely available, endoscopic straight instruments. For thoracic surgeons with experience in minimally invasive surgery, it is feasible to transit from multiple-port VATS to SIC approach because of the similar operating angle between the instruments. No new instrument is needed during the transition phase. Our experiences suggest that the learning curve is approximately 20 cases. Compared with conventional multiple-port VATS, this procedure shown comparable operative time, perioperative blood loss, and rate of conversion to open thoracotomy (data not shown).

In conclusion, we observed the safety and efficacy of a SIC three-port thoracoscopic approach in a left upper lobe sleeve resection. This approach will be a noteworthy choice in VATS.

## Acknowledgment

Thanks for this patient who consented to publish his case.

## Supplementary Material

Supplemental Digital Content

## Supplementary Material

Supplemental Digital Content
